# Laboratory and Clinical Values of the Neutrophil-to-Lymphocyte Ratio in Women With Hyperemesis Gravidarum: A Systematic Review and Meta-Analysis

**DOI:** 10.1155/jp/4872025

**Published:** 2025-09-30

**Authors:** Miguel Cabanillas-Lazo, Patricio Castro-Suárez, Sandra Uriol-Alvino, Manuel Fernandez-Navarro, Frank Mayta-Tovalino

**Affiliations:** ^1^Academic Department, NEMECS Research Group: Neurosciences, Metabolism, Clinical and Healthcare Effectiveness, Universidad Científica del Sur, Lima, Peru; ^2^Department of Gynecology and Obstetrics, Hospital de Apoyo Santa Rosa, Lima, Peru; ^3^Department of the San Fernando Scientific Society, Lima, Peru; ^4^Academic Department, San Fernando School of Medicine, Universidad Nacional Mayor de San Marcos, Lima, Peru; ^5^Department of Scientia Clinical and Epidemiological Research Institute, Trujillo, Peru; ^6^Academic Department, Vice-Rector's Office for Research, Universidad San Ignacio de Loyola, Lima, Peru

**Keywords:** diagnosis, hyperemesis gravidarum, neutrophil-to-lymphocyte ratio, NLR

## Abstract

**Introduction:**

Hyperemesis gravidarum (HG), which is characterized by severe nausea and vomiting, can lead to maternal complications and adverse fetal outcomes. The neutrophil-to-lymphocyte ratio (NLR) is a potentially simple and cost-effective marker for detecting this condition. The aim of this study was to consolidate the current evidence regarding the utility of NLR in diagnosing and assessing the severity of HG.

**Methods:**

A systematic search of Scopus, PubMed, Web of Science, Embase, and Google Scholar was conducted before March 2024. The selected articles were reviewed. Analytical cross-sectional studies reporting NLR values in patients with HG were included. Two independent authors reviewed the articles and assessed them for bias. A meta-analysis with random effects was conducted to compare NLR values between HG and healthy patients and to evaluate its association with the severity of symptoms assessed through the modified Pregnancy-Unique Quantification of Emesis, such as ketonuria and C-reactive protein levels. The GRADE system determined the certainty of the evidence.

**Results:**

Fifteen studies were included, predominantly case–control. Pooled analysis revealed a significant elevation in NLR among patients with HG compared with healthy pregnant women (MD: 1.76; 95% CI: 1.15–2.37; *I*^2^ = 98%). NLR levels were elevated in moderate (MD: 1.15; 95% CI: 0.08–2.22; *I*^2^ = 91%) and severe cases (MD: 1.25; 95% CI: 0.40–2.11; *I*^2^ = 84%) compared with mild presentations. Evidence ranged from moderate to low.

**Discussion:**

With low certainty, the mean NLR was higher in patients with HG than in healthy pregnant women, with moderate certainty regarding severity. These findings suggest the potential utility of NLR; however, further research on neonatal and long-term outcomes is needed.

**Precis:**

These results indicate that NLR could be useful, but additional studies are necessary to understand its impact on neonatal and long-term outcomes

## 1. Introduction

Hyperemesis gravidarum (HG) is a condition whose symptoms, according to consensus, begin before 16 weeks of gestational age and are characterized by nausea, intense vomiting, an inability to eat, and great limitation in daily activities [1]. It is characterized by not responding to dietary modifications or antiemetic treatment [2]. The overall prevalence of this condition is 1.1%, although this value varies due to different diagnostic criteria [3]. HG can cause water and electrolyte imbalance, anxiety, and fatal consequences, such as Wernicke's encephalopathy and vitamin K deficiency [3, 4]. In newborns exposed to mothers with HG, it is associated with premature delivery and low birth weight [5, 6]. In addition, it has been reported that in the long term, it can cause problems in the neurological development and mental health of children [7].

The etiopathogenesis of HG is not fully understood, but it is believed to involve genetic, hormonal, gastrointestinal, immunological, and psychological factors [7]. During pregnancy, the sympathetic nervous system is activated to supply oxygen and nutrients to the fetus. Granulocytes, natural killer (NK) lymphocytes, and extrathymic T cells present adrenergic receptors, which increase myelopoiesis after stimulation. However, the expression of these receptors in T and B lymphocytes is lower [8]. In addition, elevated serum levels of TNF-*α*, a cytokine derived from Th1 and Th2 lymphocytes, have been reported in patients with HG. Overactivation of these cells and high cytokine concentrations may be involved in HG [9].

A wide variety of definitions used in clinical trials have been reported, which makes it difficult to interpret their results [10]. Therefore, several biomarkers have been investigated that could serve as diagnostic markers or are associated with severity; however, many of these are difficult to access [11]. In this sense, the neutrophil-to-lymphocyte ratio (NLR) is a simple and inexpensive marker that could serve for the diagnosis and evaluation of HG severity because it is also associated with various other gynecological pathologies, such as endometriosis, ovarian cancer, and preeclampsia [12].

Therefore, this systematic review with meta-analysis was aimed at summarizing current evidence on the ability of NLR to diagnose and assess HG severity.

## 2. Materials and Methods

This study was reported according to the PRISMA [13]. The protocol was registered in PROSPERO under CRD42023463249. The PICO question for our systematic search was that, in patients with HG, does the NLR have clinical value to differentiate them from healthy pregnancies and to assess the severity and prognosis of this condition?

### 2.1. Eligibility Criteria

Studies were selected based on the following criteria: The study included pregnant female patients, reported NLR values in patients with HG, and consisted of analytical observational studies, such as cross-sectional, case–control, or cohort studies. Excluded from the study were narrative and systematic reviews, nonhuman studies, case reports, conference abstracts, and letters. The literature search was conducted in PubMed, Embase, Scopus, Web of Science, and Google Scholar up to March 2024. The search strategy was tailored for each database (Supporting Information: S1). There were no restrictions on language or publication dates. Additionally, we reviewed the bibliographies of the included studies to identify further relevant articles.

### 2.2. Study Selection

The electronic search results were imported into Endnote X9 for initial organization. Duplicate records were identified and subsequently transferred to Rayyan for further screening and management. A peer review was conducted by two reviewers (S.U-A. and P.C-S.), with any disagreements resolved by consensus and consultation with a third director (M.C-L.). The reviewers independently assessed the inclusion criteria by thoroughly reading the full texts of potentially relevant studies and resolving any discrepancies through consensus. The complete list of excluded articles can be found in Supporting Information: S2.

### 2.3. Data Extraction

Two authors (S.U-A. and P.C-S.) independently extracted data using a standardized form, resolving any disagreements through consensus and, if necessary, with the input of a third author (M.C-L.). The extracted data included the study title, first author, publication year, study design, country of study, number of participants, sex, age, sample time, mean or median NLR, follow-up crude and adjusted association measures, type of outcome, and its definition. If additional information was needed, the corresponding author was contacted via email.

### 2.4. Risk of Bias Assessment

The quality of analytical studies was assessed by two authors (S.U-A. and P.C-S.) [14] using the Newcastle Ottawa Scale (NOS). This tool evaluates the quality of published nonrandomized studies based on three main criteria: selection, comparability, and outcome/exposure, with each criterion containing subitems scored with stars. Studies with scores of 6 or higher were considered to have a low risk of bias (high quality), scores of 4–5 indicated a moderate risk of bias, and scores below 4 suggested a high risk of bias. Additionally, the quality of cross-sectional studies was evaluated using the Joanna Briggs Institute (JBI) tool, which includes eight criteria: clear inclusion and exclusion criteria, description of study subjects and setting, use of a valid and reliable method to measure exposure, standard criteria for measuring the condition, identification of confounding factors, strategies to address confounding factors, use of a valid and reliable method to measure outcomes, and appropriate statistical analysis. JBI risk of bias results were classified as low (≥ 70% positive answers), moderate (50%–69% positive answers), or high (< 49% positive answers).

### 2.5. Outcomes

Our outcomes included the evaluation of NLR, which was calculated as a simple ratio between the neutrophil and lymphocyte counts measured in peripheral blood, comparing healthy pregnant women and HG patients defined as persisting nausea and vomiting, presence of ketonuria, and loss of > 5% of body weight; (2) severity of symptoms expressed with modified Pregnancy-Unique Quantification of Emesis and Nausea (PUQE) where < 7 points means mild symptoms; 7–12 points, moderate symptoms; and > 12 points, severe symptoms [15]; (3) prognosis (maternal and fetal complications); and (4) laboratory measurements (C-reactive protein [CRP] and ketonuria). No studies have evaluated NLR with any prognostic variables.

### 2.6. Data Analysis

A meta-analysis was planned for each outcome, with data analysis conducted using the R programming language. Mean differences (MDs) and their 95% confidence intervals (95% CIs) were pooled using the inverse variance method and the random-effects model. The variance between studies (*τ*^2^) was estimated using the DerSimonian–Laird estimator, and heterogeneity among studies was assessed using the *I*^2^ statistic. For meta-analyses involving correlation data, the metacor function from the meta v5.5 R package was used. Pooled correlation coefficients with 95% CI were calculated using the random-effects model with the inverse variance weighting method and the restricted maximum likelihood method estimator for between-study variance. Before analysis, correlation coefficients were transformed into Fisher's *z*, unless the included studies had very large sample sizes. This transformation was performed automatically by the metacor function with the sm argument set to “ZCOR.” Different types of correlations were not pooled together, as Pearson's product–moment correlation is used for linear relationships between two continuous variables, while Spearman's rank correlation is used for monotonic but nonlinear relationships. In our meta-analysis, heterogeneity was defined as low if *I*^2^ < 30%, moderate if *I*^2^ = 30%–60%, and high if *I*^2^ > 60%. A subgroup analysis was performed based on case–control match. Finally, we performed a leave-one-out publication bias analysis, using the Paule–Mandel estimator and excluded studies with non–low risk of bias. This decision was made because the DerSimonian–Laird estimator underestimates true heterogeneity when *τ*^2^ is large [16, 17]. To interpret the correlation coefficients, we evaluated the framework presented by Schober et al., where a coefficient of 0.00–0.09 was considered negligible, 0.10–0.39 as weak, 0.40–0.69 as moderate, and 0.70–0.89 as strong correlation [18].

### 2.7. Evidence Certainty Assessment

Two authors (C.C.B. and C.Q.V.) independently assessed the certainty of our pooled results and qualitative synthesis using the Grading of Recommendations, Assessment, Development, and Evaluation (GRADE) approach for continuous outcomes and prognostic factors [19, 20]. This assessment considers five domains: study limitations (risk of bias in the included studies), imprecision (sample size and confidence interval), indirectness (generalizability), inconsistency (heterogeneity), and publication bias. It also accounts for the large magnitude of an effect, dose–response gradient, and the effect of plausible residual confounding, as outlined in the GRADE handbook [21]. We adapted the GRADE criteria to our results (Table S2). The certainty of the evidence was characterized as high, moderate, low, or very low. To evaluate the imprecise criteria in the correlation of coefficients, we interpret it as imprecise if the confidence interval passes weak and negligible correlation values. Publication bias was assessed using funnel plots and Egger's and Begg's tests.

## 3. Results

### 3.1. Study Selection

**Figure 1:**
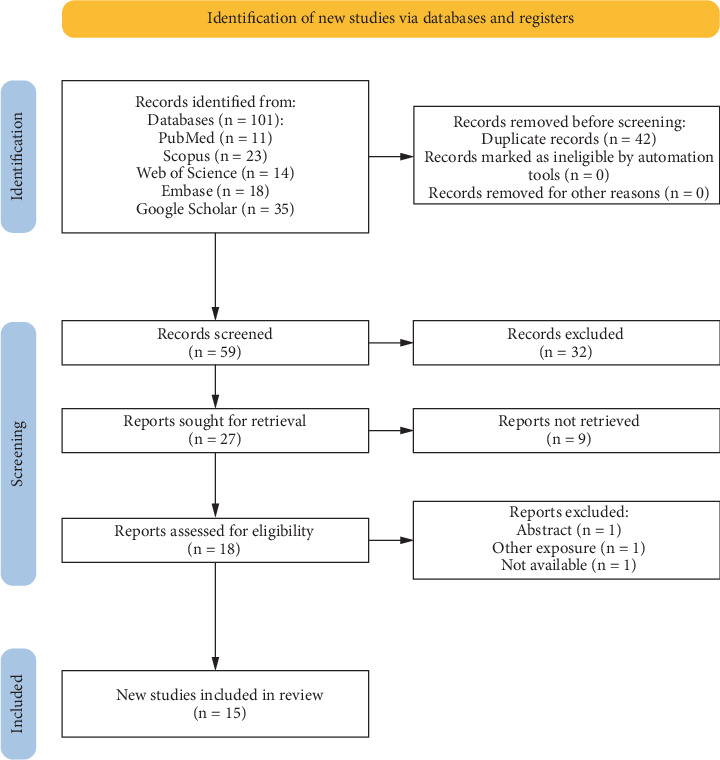
PRISMA flowchart of the studies included.

We identified 101 studies through our systematic search. After removing duplicates, we screened 59 studies and ultimately included 15 articles (Figure 1) [22–36].

### 3.2. Characteristics of Studies

All except one were case–control studies. The total number of participants was 3900 (2276 HG patients and 1624 healthy pregnant women). The mean ages of pregnant women with HG ranged from 26.02 to 29.41 years. The characteristics of the studies are summarized in Table 1.

### 3.3. Risk of Bias Assessment

Fourteen case–control studies were evaluated using the NOS, all of which were cataloged as “low risk of bias,” and eight of them got the lowest score (6/9) because insufficient or absent case–control matching was reported by the authors. The assessment of Besser et al. cross-sectional study resulted in “low risk of bias” in 100% of positive answers to the JBI questionnaire (Supporting Information: S3).

### 3.4. Patients With HG and Healthy Pregnant Women

**Figure 2:**
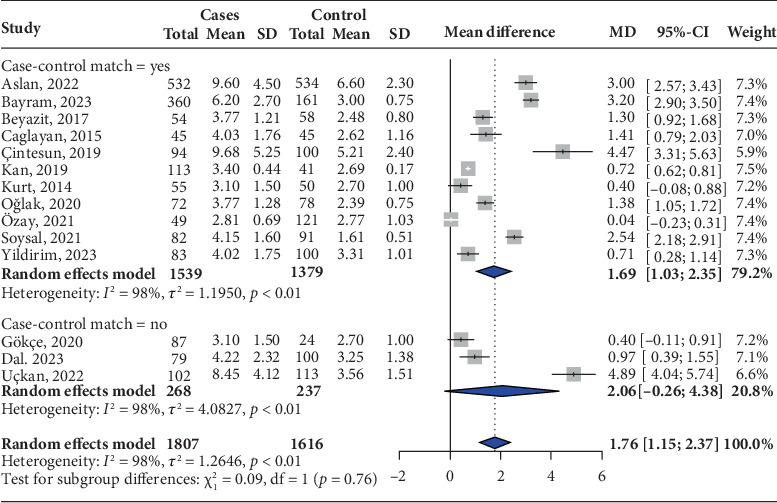
Mean difference in the neutrophil-to-lymphocyte ratio (NLR) between patients with hyperemesis gravidarum and healthy controls according to reported match.

Among the 15 studies that met our inclusion criteria, 14 were included in the pooled analysis. A total of 3423 participants were selected, with 1807 assigned to the HG group and 1616 to the control group. The pooled analysis revealed a significant increase in the NLR, accompanied by high heterogeneity (MD: 1.76; 95% CI: 1.15, 2.37; *I*^2^ = 98%) (Figure 2).

In the subgroup analysis comparing studies with matched patients and healthy pregnant controls, a significantly elevated NLR was observed in patients with HG (MD: 1.69; 95% CI: 1.03–2.35; *I*^2^ = 98%). Conversely, in studies without matching procedures, the difference in NLR was not statistically significant (MD: 2.06; 95% CI: −0.26 to 4.38; *I*^2^ = 98%) (Figure 2).

Regarding our sensitivity analysis, when single studies were sequentially removed, no significant variation in the pooled MD was observed (between 1.54 and 1.90) (Supporting Information: S4). In addition, no significant variation was observed using the Paule–Mandel estimator (MD: 1.78; 95% CI: 0.98, 2.58; *I*^2^ = 98%) (Supporting Information: S5). Finally, when we excluded studies with moderate and high risk of bias, no significant variation was observed (MD: 1.46; 95% CI: 0.76, 2.17; *I*^2^ = 96%) (Supporting Information: S6). This suggests that the results of the meta-analysis were stable.

### 3.5. Symptom Severity

**Figure 3:**
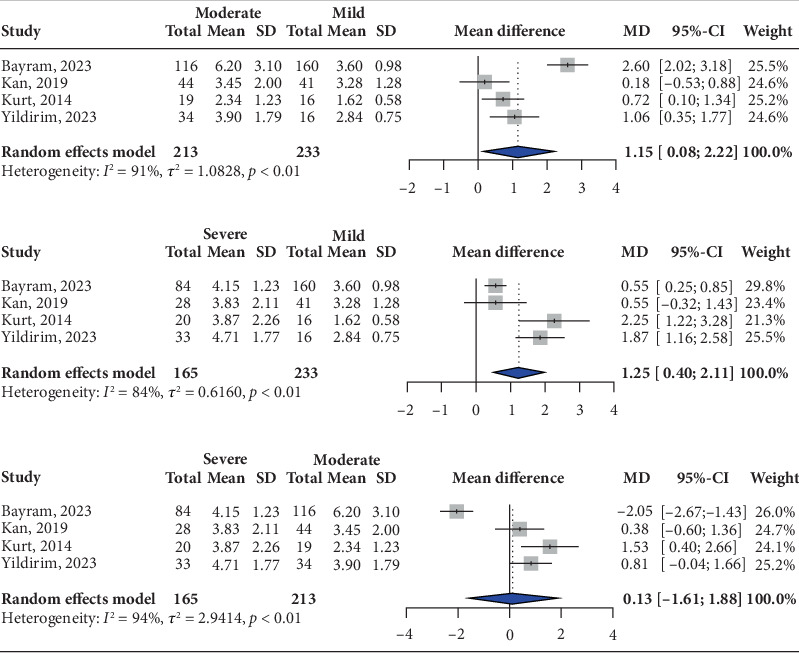
Mean difference in the neutrophil-to-lymphocyte ratio (NLR) between severity groups in hyperemesis gravidarum patients. (a) Moderate and mild hyperemesis. (b) Severe and mild hyperemesis. (c) Severe and moderate hyperemesis.

By comparing patients with moderate and mild nausea and vomiting, a total of 446 participants were pooled, with a significant increase in moderate patients with high heterogeneity (MD: 1.15; 95% CI: 0.08, 2.22; *I*^2^ = 91%) (Figure 3a). Regarding our sensitivity analysis, when single studies were removed sequentially, significant variations in the pooled 95% CI were observed (Supporting Information: S7). No significant variation was observed using the Paule–Mandel estimator (MD: 1.15; 95% CI: 0.13, 2.17; *I*^2^ = 91%) (Supporting Information: S8).

By comparing patients with severe and mild nausea and vomiting, a total of 398 participants were pooled, with a significant increase in severe patients with high heterogeneity (MD: 1.25; 95% CI: 0.40, 2.11; *I*^2^ = 84%) (Figure 3b). Regarding our sensitivity analysis, when single studies were removed sequentially, nonsignificant variations in the pooled 95% CI were observed (Supporting Information: S9). No significant variation was observed using the Paule–Mandel estimator (MD: 1.25; 95% CI: 0.40, 2.11; *I*^2^ = 84%) (Supporting Information: S10).

By comparing patients with severe and moderate nausea and vomiting, a total of 378 participants were pooled, with a nonsignificant increase in the severe group with high heterogeneity (MD: 0.13; 95% CI: −1.61, 1.88; *I*^2^ = 94%) (Figure 3c). Regarding our sensitivity analysis, when single studies were removed sequentially, significant variations in the pooled 95% CI were observed (Supporting Information: S11). No significant variation was observed using the Paule–Mandel estimator (MD: 0.12; 95% CI: −1.41, 1.66; *I*^2^ = 94%) (Supporting Information: S12).

### 3.6. NLR and Ketonuria Levels

Two studies with 1083 patients included the calculated correlation coefficients between NLR and severity (Supporting Information: S13). The pooled results showed a significant correlation (*z* = 0.70; 95% CI: 0.19, 1.21; *I*^2^ = 98%) with Spearman's correlation transformed (*r* = 0.61; CI: 0.19, 0.84).

### 3.7. NLR and CRP

Two studies, with 141 patients, included calculated correlation coefficients between NLR and severity (Supporting Information: S14). The pooled results showed a nonsignificant correlation (*z* = 0.67; 95% CI: −0.65, 0.19, 1.98; *I*^2^ = 98%) with Pearson's correlation transformed (*r* = 0.58; CI: −0.57, 0.96).

### 3.8. Publication Bias

Begg's test (*z* = 0.82, *p* = 0.4115) and Egger's test (*t* = 2.04, *p* = 0.0635) suggested that there may be some evidence of publication bias, and the funnel plot showed asymmetry in MD between HG cases and healthy pregnant women (Supporting Information: S15).

### 3.9. Evidence Certainty

For patients with HG and healthy pregnant women, the certainty of the included evidence was assessed to be low (Table 2). Our evaluation began from a high level of certainty due to the inclusion of comparative observational studies. We have downgraded the manuscript due to the high heterogeneity of the studies and possible publication bias. Regarding the severity of nausea and vomiting assessed using PUQE, we began the assessment with a high level of certainty because all the included studies were observational in nature. The difference in NLR between low compared to moderate and severe symptoms has moderate certainty due to the high heterogeneity of the studies; however, the difference in NLR between severe and moderate symptoms has low certainty due to high heterogeneity and imprecision. Finally, regarding laboratory measurements, we began the assessment with a high level of certainty since all the included studies were observational. The correlation between NLR and ketonuria and CRP levels is highly uncertain because of imprecision and high heterogeneity.

## 4. Discussion

The present systematic review with meta-analysis included 3423 participants, showing significant findings regarding NLRs, revealing a significant increase in the mean NLR in patients with HG, compared with healthy patients, showing a positive association between the condition and systemic inflammatory response, with a high level of certainty. However, it is important to highlight the notable heterogeneity among the studies included in our review. Additionally, we observed a notable trend in patients with moderate or severe symptoms (according to the PUQE scale) who showed significantly higher NLR levels compared with those with mild nausea and vomiting. This difference suggests a relationship between the severity of HG symptoms (assessed with the PUQE scale) and the magnitude of the inflammatory response, as measured by NLR.

Our pooled analysis revealed that the average NLR was higher in patients with HG than in healthy individuals. The sensitivity analyses showed that these results remained consistent and were not influenced by studies reporting case–control matching. These findings are consistent with those of previous studies that evaluated NLR in other gynecological and obstetric inflammatory disorders. For example, Fatemeh et al. in a study that included 18 articles observed a significant increase in NLR in patients with endometriosis compared with healthy patients (SMD = 0.79, 95%CI = 0.33–1.25, *p* < 0.001) [37]. Kamran et al. reported that a meta-analysis of six studies revealed higher NLR values in patients with gestational diabetes compared with those without the disease (SMD = 0.48, 95%CI = 0.25–0.71) [38]. Likewise, Shokoufeh et al. included 15 articles showing elevated NLR values in patients with adnexal torsion compared with those with any other adnexal mass (SMD = 1.06, 95%CI = 0.67–1.45, *p* < 0.001) [39]. The mechanism that could explain this relationship between NLR and HG lies in the high concentrations of cytokines that pregnant women may have, resulting in an exaggerated or dysregulated inflammatory response in the body.

Systematic reviews that address the relationship between NLR and pathological severity are limited. Kang et al. conducted a meta-analysis reporting eight studies that showed elevated NLR levels in patients with severe preeclampsia compared with healthy women (MD: 1.92, 95% CI [1.31, 2.53]); they also identified seven studies reporting increased NLR values in women with severe preeclampsia compared with mild cases (MD: 1.12, 95% CI [0.69, 1.56]) [40]. The higher number of adrenergic receptors in granulocytes compared with lymphocytes may cause neutrophilia in this condition.

The certainty of evidence for NLR among patients with HG was low, due to high heterogeneity and imprecision, highlighting the need for prospective studies with a large sample size to enhance result robustness. The observed heterogeneity in our meta-analysis may stem from variations in blood sampling timing and gestational age, potentially influencing result interpretation; thus, expanding participant numbers in future research could yield a more precise understanding of the NLR-HG association. Furthermore, we recommend conducting studies in different parts of the world since our results are mainly from the Turkish population, and there is evidence of genetic compromise in HG [41]. Conducting research to evaluate the diagnostic performance of NLR as a diagnostic test for HG would be beneficial. By determining the sensitivity and specificity of NLR for the detection of this condition, medical professionals can use it as an effective tool for the early diagnosis and precise identification of patients with HG. This could improve disease management and treatment, resulting in better outcomes for both the patients and their fetuses. Furthermore, the absence of studies addressing the short- and long-term maternal–fetal repercussions of elevated NLR in patients with HG underscores the need for more comprehensive research in this area. Identifying the effects of increased NLR on maternal and fetal health could provide valuable insights to enhance the clinical management of HG and develop more effective intervention strategies. The presence of suspected publication bias in the results for patients and healthy subjects, the irregular dispersion, and the absence of small studies with negative or neutral effects could indicate that studies with nonsignificant results and small sample sizes are not published. Therefore, we must be cautious when interpreting our results, and we encourage promoting the publication of null or negative results to ensure an adequate evidence base.

The NLR, as a marker of systemic inflammation, has shown valuable clinical utility because of its ease of measurement, low cost, and availability for routine exams [42]. This indicator, in addition to the current clinical and laboratory criteria [43], could help differentiate between healthy individuals and those with HG, as well as assess the severity of symptoms and inflammatory markers in patients with this condition [44]. Our findings revealed that NLR was significantly higher in patients with HG than in healthy individuals and was even more elevated in those with severe HG than in mild cases. This difference in NLR levels may have significant implications for defining therapeutic approaches, whether for outpatient management or hospital intervention, although this is still not conclusive.

We conducted a comprehensive and unrestricted systematic search across multiple databases, regardless of language or publication year, to ensure a thorough examination of the available evidence. Furthermore, we assessed the certainty of our results using the GRADE system, thereby enhancing the reliability and credibility of our findings. This systematic review has several limitations that should be considered. First, the researchers excluded Chinese databases from the bibliographic search, potentially limiting the comprehensiveness of the literature reviewed. All studies included in this review were either retrospective or cross-sectional in nature, which could introduce biases and affect the robustness of the findings. Finally, high and significant heterogeneity was observed among the selected studies, which may have affected the interpretation of the results. However, it is important to acknowledge the strengths of our study as well.

## 5. Conclusion

The mean NLR was found to be higher in patients with HG compared to healthy pregnant women. Additionally, with low to moderate certainty, we determined that NLR could be an indicator of the severity of PUQE, ketonuria, and CRP levels. Future prospective studies in diverse populations and the development of diagnostic models incorporating NLR are necessary.

## Figures and Tables

**Table 1:** Characteristics of the included studies (*n* = 15).

**Author, year**	**Country**	**Study design**	**N** ** HG**	**N** ** controls**	**Match HG control**	**Patients' source**	**Age, mean (SD)**	**Follow-up time**	**Outcome**	**Cut point NLR**	**Gestational age (weeks), mean (SD)**	**Blood sample time**
Aslan, 2022 [22]	Turkey	Case–control	532	534	Age	HG: Inpatient clinic Controls: NR	Controls: 25.9 (4.8) HG: 26.3 (4.1)	NR	Diagnostics Prognostics (severity of ketonuria)	NR	6–13	Day of admission
Bayram, 2023 [23]	Turkey	Case–control	360	161	Gestational age	NR	27.6 (16–40)^a^	NR	Diagnostics Prognostics (PUQE score)	NR	HG patients: 9 (5–20)^a^ Control: 9 (1–19)^a^	During their hospitalization
Beser, 2023 [24]	Turkey	Cross-sectional	469	NR	NR	HG: Inpatient clinic	27.5 (5.4)	NR	Prognostics (severity of ketonuria)	NR	9.4 (2.6)	During their hospitalization
Beyazit, 2017 [25]	Turkey	Case–control	54	58	Age and gestational age	NR	HG: 25 (17–43)^a^ Controls: 27 (19–44)^a^	NR	Diagnostics Prognostics (CRP, ESR, WBC)	2.38	HG patients: 9 (7–13)^a^ Control: 9 (7–12)^a^	NR
Caglayan, 2015 [26]	Turkey	Case–control	45	45	Ethnicity, race, age, body mass index (BMI), gravidity, and gestational week	HG: OBGYN department Controls: NR	HG: 26.02 (5.17) Controls: 27.76 (5.28)	NR	Diagnostics	NR	HG patients: 8 (1–13)^a^ Control: 10 (1–14)^a^	NR
Çintesun, 2019 [27]	Turkey	Case–control	94	100	Gestational age	NR	HG: 26 (17−40)^a^ Controls: 27 (18−41)^a^	NR	Diagnostics Prognostics (severity of ketonuria)	4.11	6–13^b^	NR
Dal, 2023 [28]	Turkey	Case–control	79	100	NR	HG: Outpatient/inpatient clinic Controls: NR	HG: 27.85 (5.24) Controls: 29.29 (6.30)	NR	Diagnostics Prognostics (severity of ketonuria)	NR	HG patients: 9.7 (2.2) Control: 9.3 (2.2)	NR
Gökçe, 2020 [29]	Turkey	Case–control	87	24	NR	HG: Outpatient clinic Controls: NR	25.4 (5.0)	NR	Diagnosis Severity (CRP, leukocyte count)	NR	4–27^b^	Day of admission
Kan, 2019 [30]	Turkey	Case–control	113	49	Age and gestational age	HG: Emergency department Controls: NR	Controls: 25.95 (5.57) HG: 26.3 (4.87)	NR	Diagnostics Prognostic (PUQE score, VAS)	2.78	1–16^b^	Day of admission
Kurt, 2014 [31]	Turkey	Case–control	55	50	Gestational age	NR	HG: 29 (2.8) Controls: 30 (3.1)	NR	Diagnostics Prognostics (PUQE score)	NR	HG patients: 9.4 (3.0) Control: 8.8 (2.5)	NR
Oğlak, 2020 [32]	Turkey	Case–control	72	78	Age, body mass index, gravida, parity, and gestational age	HG: Inpatient clinic Controls: NR	HG: 24 (17–40)^a^ Controls: 27 (18–40)^a^	NR	Diagnostics	NR	HG patients: 10 (7–14)^a^ Control: 9 (7–13)^a^	Day of admission
Özay, 2021 [33]	Cyprus	Case–control	49	121	Gestational age	NR	Controls: 29.22 (6.00) HG: 28.10 (4.87)	NR	Diagnostics	NR	HG patients: 7 (6–13)^a^ Control: 7 (6–12)^a^	NR
Soysal, 2021 [34]	Turkey	Case–control	82	91	Ethnicity, race, gravida, and pregnancy week	HG: Inpatient clinic Controls: NR	HG: 27.9 (19–40)^a^ Controls: 26.8 (19–36)^a^	NR	Diagnostics Prognostics (severity of ketonuria)	NR	HG patients: 9.57 (7.00–11.57)^a^ Control: 9.43 (6.43–11.29)^a^	NR
Uçkan, 2022 [35]		Case–control	102	113	NR	HG: Inpatient clinic Controls: NR	Controls: 29.49 (18–42)^a^ HG: 29.89 (18–42)^a^	NR	Diagnostics Prognostics (PUQE score)	NR	HG patients: 10.34 (6–13)^a^ Control: 10.53 (7–12)^a^	NR
Yıldırım, 2023 [36]	Turkey	Case–control	83	100	Gestational age	NR	HG: 29.11 (5.04) Controls: 29.41 (5.65)	NR	Diagnostics Prognostics (PUQE score)	NR	HG patients: 8.88 (1.63) Control: 8.23 (1.10)	NR

Abbreviations: CRP, C-reactive protein; ESR, erythrocyte sedimentation rate; HG, hyperemesis gravidarum; NR, not reported; PUQE, Pregnancy-Unique Quantification of Emesis and Nausea questionnaire; WBC, white blood cell count.

^a^Median (range).

^b^Range.

**Table 2:** GRADE summary of findings.

**Outcomes**	**No. of participants (studies)**	**The certainty of the evidence (GRADE)**	**Anticipated absolute effects**	
			**Mean difference (MD)**	**95% CI**
Differences between patients with HG and healthy participants	3423 (14 observational studies)	⨁⨁◯◯ Low^a^^,^^c^	1.76	1.15–2.37
Difference between moderate and mild hyperemesis (PUQE)	446 (4 observational studies)	⨁⨁⨁◯ Moderate^a^	1.15	0.08–2.22
Severe and mild hyperemesis (PUQE)	398 (4 observational studies)	⨁⨁⨁◯ Moderate^a^	1.25	0.40–2.11
Severe and moderate hyperemesis (PUQE)	378 (4 observational studies)	⨁⨁◯◯ Low^a^^,^^b^	0.13	−1.61 to 1.88
Ketonuria level	1083 (3 observational studies)	⨁⨁◯◯ Low^a^^,^^d^	0.61⁣^∗^	0.19–0.84
CRP level	141 (2 observational studies)	⨁⨁◯◯ Low^a^^,^^b^	0.58⁣^∗∗^	−0.57 to 0.96

*Note:* GRADE Working Group on evidence grades. High certainty: we are very confident that the true effect lies close to that of the estimated effect. Moderate certainty: we are moderately confident in the effect estimate: the true effect is likely to be close to the estimate of the effect, but there is a possibility that it is substantially different. Low certainty: the confidence in the effect estimate is limited: the true effect may differ substantially from the estimated effect. Very low certainty: we have very little confidence in the effect estimate: the true effect is likely to substantially differ from the estimated effect.

Abbreviations: CI, confidence interval; CRP, C-reactive protein; HG, hyperemesis gravidarum; MD, mean difference; PUQE, modified Pregnancy-Unique Quantification of Emesis and Nausea.

^a^High heterogeneity between studies was detected in the meta-analysis.

^b^Confidence interval crosses the point of no effect.

^c^Possible publication bias.

^d^Imprecise confidence interval (rho Spearman < 0.39).

⁣^∗^Rho Spearman.

⁣^∗∗^*r* Pearson.

## Data Availability

The data will be available upon request to the corresponding author.
