# Supplementary material for: Laboratory and Clinical Values of the Neutrophil-to-Lymphocyte Ratio in Women With Hyperemesis Gravidarum: A Systematic Review and Meta-Analysis
Source: J Pregnancy. 2025 Sep 30;2025:4872025. doi: 10.1155/jp/4872025 (PMC12503971; doi:10.1155/jp/4872025)

**REVIEW ARTICLE**

**Laboratory and clinic values of the neutrophil-to-lymphocyte ratio in women with Hyperemesis gravidarum: A systematic review and meta-analysis**

**Supplementary Material S1. Search strategy for the databases used**

| PubMed | #1: Hyperemesis Gravidarum[MH] OR “Vomiting of Pregn*”[tiab] OR “vomiting in pregn*”[tiab] OR “hyperemesis in pregn*”[tiab] OR “hyperemesis of pregn*”[tiab] OR “Pregnancy Pernicious Vomit*”[tiab] OR “gestation hyperem*”[tiab] OR “hyperemesis during pregn*”[tiab] OR “hyperemesis gravid*”[tiab] OR “pregnancy hyperem*”[tiab]  #2: "neutrophil to lymphoc*" [all fields] OR "neutrophil-to-lymphoc*"[all fields] OR "neutrophil/lymphoc*"[all fields] OR "NLR" [all fields] OR "neutrophil lymphoc*" [all fields] OR "granulocyte lymphoc*" [all fields] OR "granulocyte to lymphoc*" [all fields] OR "granulocyte- to-lymphoc*"[all fields] OR "granulocyte/lymphoc*" OR "GLR" [all fields]  #3: #1 AND #2 |
| --- | --- |
| Scopus | #1: TITLE-ABS-KEY(“Vomiting of Pregn*” OR “vomiting in pregn*” OR “hyperemesis in pregn*” OR “hyperemesis of pregn*” OR “Pregnancy Pernicious Vomit*” OR “gestation hyperem*” OR “hyperemesis during pregn*” OR “hyperemesis gravid*” OR “pregnancy hyperem*”)  #2: ALL("neutrophil to lymphoc*" OR “neutrophil-to-lymphoc*” OR “neutrophil/lymphoc*” OR "NLR" OR "neutrophil lymphoc*" OR "granulocyte lymphoc*" OR “granulocyte to lymphoc*” OR “granulocyteto-lymphoc*” OR “granulocyte/lymphoc*” OR "GLR")  #3: #1 AND #2 |
| Web of Science | #1: TS=(“Vomiting of Pregn*” OR “vomiting in pregn*” OR “hyperemesis in pregn*” OR “hyperemesis of pregn*” OR “Pregnancy Pernicious Vomit*” OR “gestation hyperem*” OR “hyperemesis during pregn*” OR “hyperemesis gravid*” OR “pregnancy hyperem*”) OR TI=(“Vomiting of Pregn*” OR “vomiting in pregn*” OR “hyperemesis in pregn*” OR “hyperemesis of pregn*” OR “Pregnancy Pernicious Vomit*” OR “gestation hyperem*” OR “hyperemesis during pregn*” OR “hyperemesis gravid*” OR “pregnancy hyperem*”) OR AB=(“Vomiting of Pregn*” OR “vomiting in pregn*” OR “hyperemesis in pregn*” OR “hyperemesis of pregn*” OR “Pregnancy Pernicious Vomit*” OR “gestation hyperem*” OR “hyperemesis during pregn*” OR “hyperemesis gravid*” OR “pregnancy hyperem*”) OR AK=(“Vomiting of Pregn*” OR “vomiting in pregn*” OR “hyperemesis in pregn*” OR “hyperemesis of pregn*” OR “Pregnancy Pernicious Vomit*” OR “gestation hyperem*” OR “hyperemesis during pregn*” OR “hyperemesis gravid*” OR “pregnancy hyperem*”) OR KP=(“Vomiting of Pregn*” OR “vomiting in pregn*” OR “hyperemesis in pregn*” OR “hyperemesis of pregn*” OR “Pregnancy Pernicious Vomit*” OR “gestation hyperem*” OR “hyperemesis during pregn*” OR “hyperemesis gravid*” OR “pregnancy hyperem*”)  #2: TS=("neutrophil to lymphoc*" OR “neutrophil-to-lymphoc*” OR “neutrophil/lymphoc*” OR "NLR" OR "neutrophil lymphoc*" OR "granulocyte lymphoc*" OR “granulocyte to lymphoc*” OR “granulocyteto-lymphoc*” OR “granulocyte/lymphoc*” OR "GLR") OR TI=("neutrophil to lymphoc*" OR “neutrophil-to-lymphoc*” OR “neutrophil/lymphoc*” OR "NLR" OR "neutrophil lymphoc*" OR "granulocyte lymphoc*" OR “granulocyte to lymphoc*” OR “granulocyteto- lymphoc*” OR “granulocyte/lymphoc*” OR "GLR") OR AB=("neutrophil to lymphoc*" OR “neutrophil-to-lymphoc*” OR “neutrophil/lymphoc*” OR "NLR" OR "neutrophil lymphoc*" OR "granulocyte lymphoc*" OR “granulocyte to lymphoc*” OR “granulocyteto-lymphoc*” OR “granulocyte/lymphoc*” OR "GLR") OR AK=("neutrophil to lymphoc*" OR “neutrophil-to- lymphoc*” OR “neutrophil/lymphoc*” OR "NLR" OR "neutrophil lymphoc*" OR "granulocyte lymphoc*" OR “granulocyte to lymphoc*” OR “granulocyteto-lymphoc*” OR “granulocyte/lymphoc*” OR "GLR") OR KP=("neutrophil to lymphoc*" OR “neutrophil-to- lymphoc*” OR “neutrophil/lymphoc*” OR "NLR" OR "neutrophil lymphoc*" OR "granulocyte lymphoc*" OR “granulocyte to lymphoc*” OR “granulocyteto-lymphoc*” OR “granulocyte/lymphoc*” OR "GLR")  #3: #1 AND #2 |
| Google Scholar | (“Hyperemesis Gravidarum”) Furthermore, ('neutrophil lymphocyte ratio' OR nlr) |
| Embase | ('hyperemesis gravidarum'/exp OR 'hyperemesis gravidarum' OR 'excessive vomiting in pregnancy' OR 'gestation hyperemesis' OR 'hyperemesis during pregnancy' OR 'hyperemesis in pregnancy' OR 'hyperemesis of pregnancy' OR 'intractable vomiting of pregnancy' OR 'pernicious vomiting in pregnancy' OR 'pernicious vomiting of pregnancy' OR 'pregnancy hyperemesis') AND (“neutrophil lymphocyte ratio”)/exp OR “nlr (lymphocyte)' OR 'neutrophil lymphocyte ratio' OR 'neutrophil to lymphocyte ratio' OR 'neutrophil/lymphocyte ratio') |

**Supplementary Material S2. Reasons for exclusion of full-text documents evaluated**

| **Title** | **Author** | **Reason for exclusion** |
| --- | --- | --- |
| Relationship between novel inflammatory markers and hyperemesis gravidarum | Çift, T. | Abstract |
| Role of the systemic inflammatory index in determining the length of hospital stay among patients with hyperemesis gravidarum | Doğru, Ş | Other exposure |
| Relationship between CRP, IL-6, NLR, and PLR levels in hyperemesis gravidarum | Li, Wei | Not available |

**Supplementary Material S3. Risk of bias of included studies**

| Study (Case control) | Selection (max 4*) | | | | Comparability (max 2*) | Exposure (max 3*) | | |  |  |
| --- | --- | --- | --- | --- | --- | --- | --- | --- | --- | --- |
|  | **Is the case definition adequate?** | **Representativeness of the cases** | **Selection of controls** | **Definition of controls** | **Comparability of cases and controls based on the design or analysis** | **Ascertainment of exposure** | **The same method of ascertainment for cases and controls** | **Non-Response rate** | **Score** | **Risk of bias** |
| Aslan, 2022 | * | * |  | * |  | * | * | * | 6 | Low risk of bias |
| Bayram, 2023 | * | * |  | * |  | * | * | * | 6 | Low risk of bias |
| Beyazit, 2017 | * | * |  | * | * | * | * | * | 7 | Low risk of bias |
| Caglayan, 2015 | * | * |  | * | ** | * | * | * | 8 | Low risk of bias |
| Çintesun, 2019 | * | * |  | * |  | * | * | * | 6 | Low risk of bias |
| Gökçe, 2020 | * | * |  | * |  | * | * | * | 6 | Low risk of bias |
| Kan, 2019 | * | * |  | * | * | * | * | * | 7 | Low risk of bias |
| Kurt, 2014 | * | * |  | * |  | * | * | * | 6 | Low risk of bias |
| Dal, 2023 | * | * |  | * |  | * | * | * | 6 | Low risk of bias |
| Oğlak, 2020 | * | * |  | * | ** | * | * | * | 8 | Low risk of bias |
| Özay, 2021 | * | * |  | * |  | * | * | * | 6 | Low risk of bias |
| Soysal, 2021 | * | * |  | * | ** | * | * | * | 8 | Low risk of bias |
| Uçkan, 2022 | * | * |  | * |  | * | * | * | 6 | Low risk of bias |
| Yildirim, 2023 | * | * |  | * |  | * | * | * | 6 | Low risk of bias |

| **JBI questionnaire for analytical cross-sectional studies** | **Beser, 2022** |
| --- | --- |
| Are the inclusion criteria clearly defined in the sample? | Yes |
| Were the study subjects and settings described in detail? | Yes |
| Was the exposure measured in a valid and reliable manner? | Yes |
| Are objective, standard criteria used for the measurement of the condition? | Yes |
| Were confounding factors identified? | Yes |
| Were strategies to deal with confounding factors | Yes |
| Were the outcomes measured in a valid and reliable manner? | Yes |
| Was the statistical analysis appropriate? | Yes |

**Supplementary Material S4. Leave-one-out sensitivity analysis between patients with hyperemesis gravidarum and healthy controls**


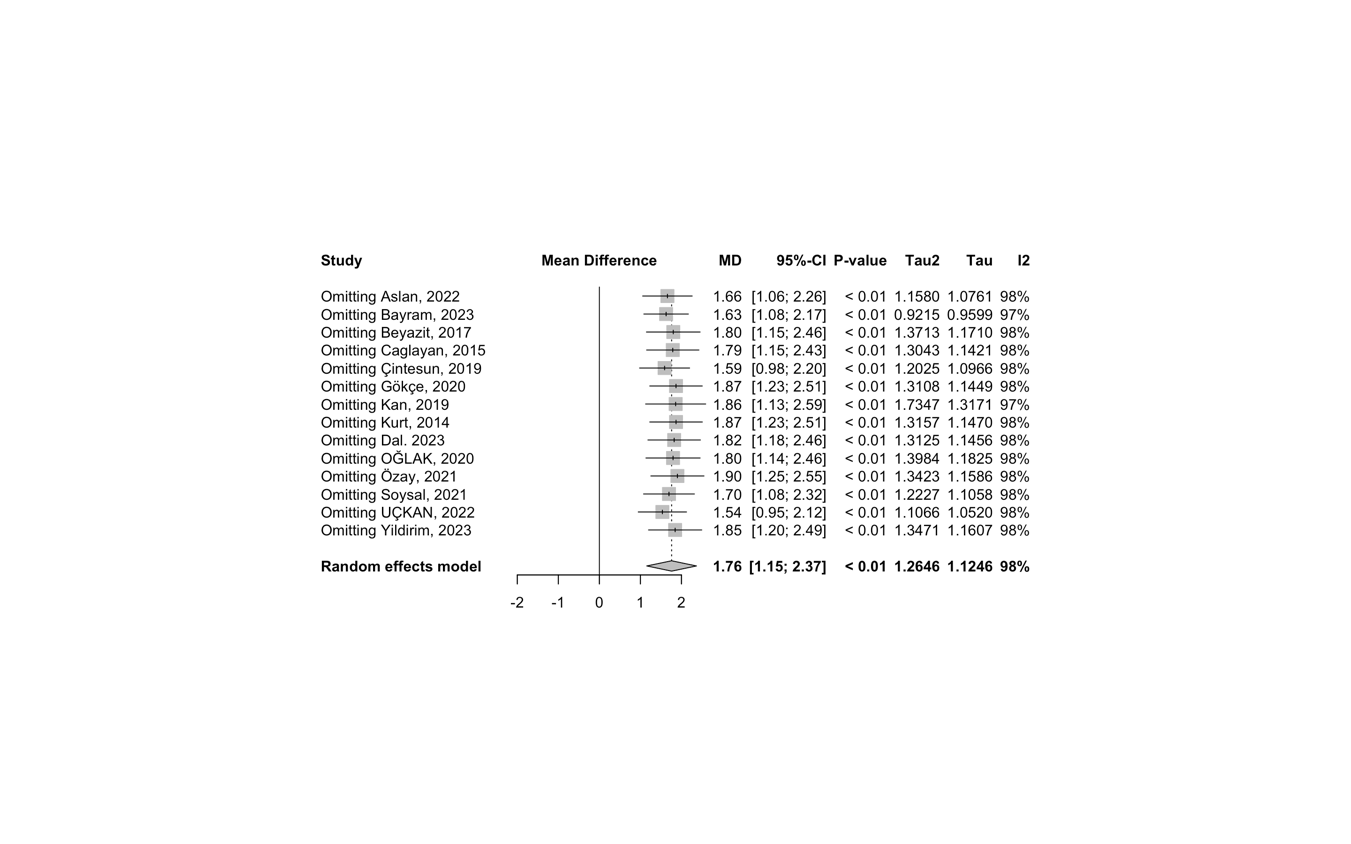


**Supplementary Material S5. Meta-analysis using Paule-Mandel estimator between patients with hyperemesis gravidarum and healthy controls**


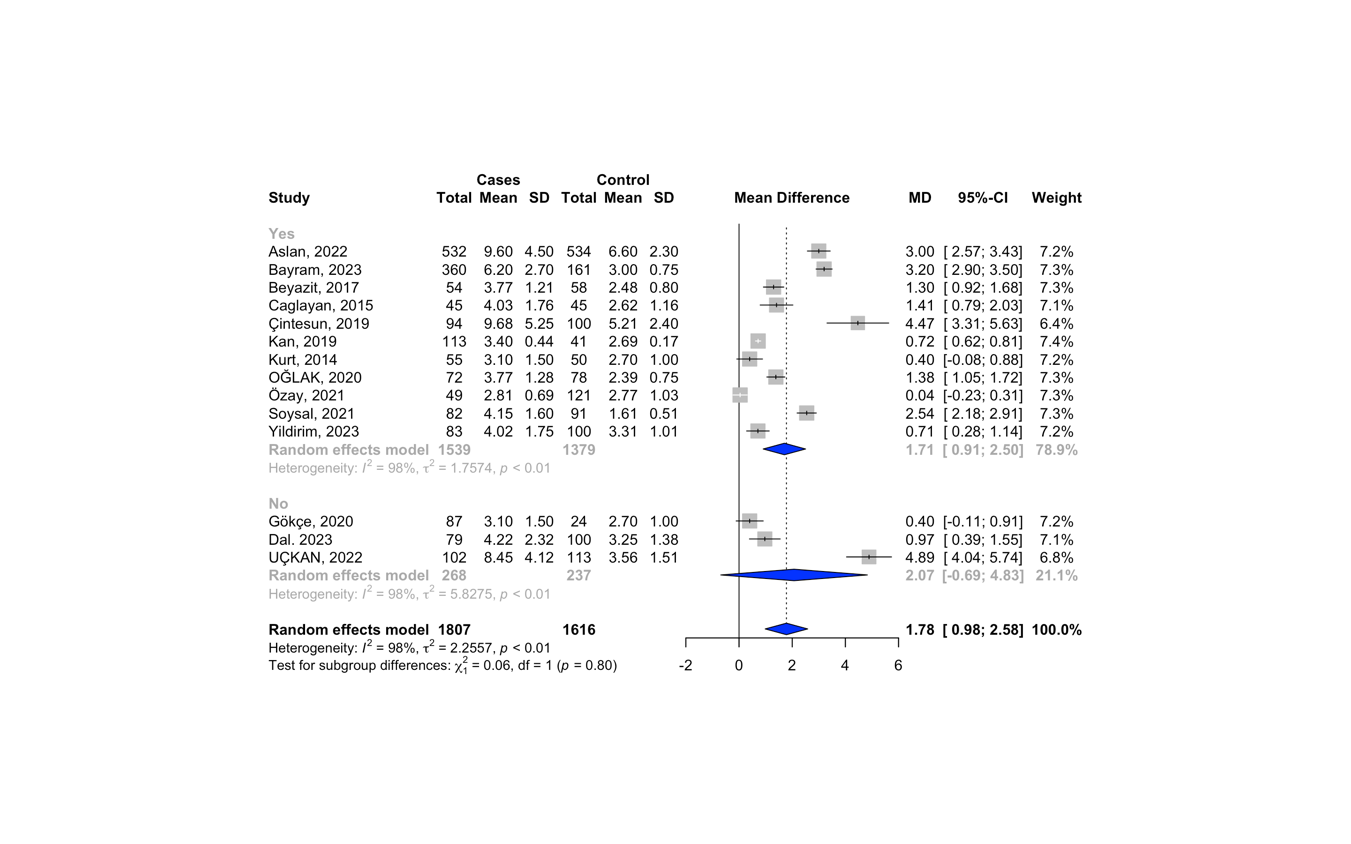


**Supplementary Material S6. Meta-analysis excluding studies with moderate and high risk of bias regarding patients with hyperemesis gravidarum and healthy controls**


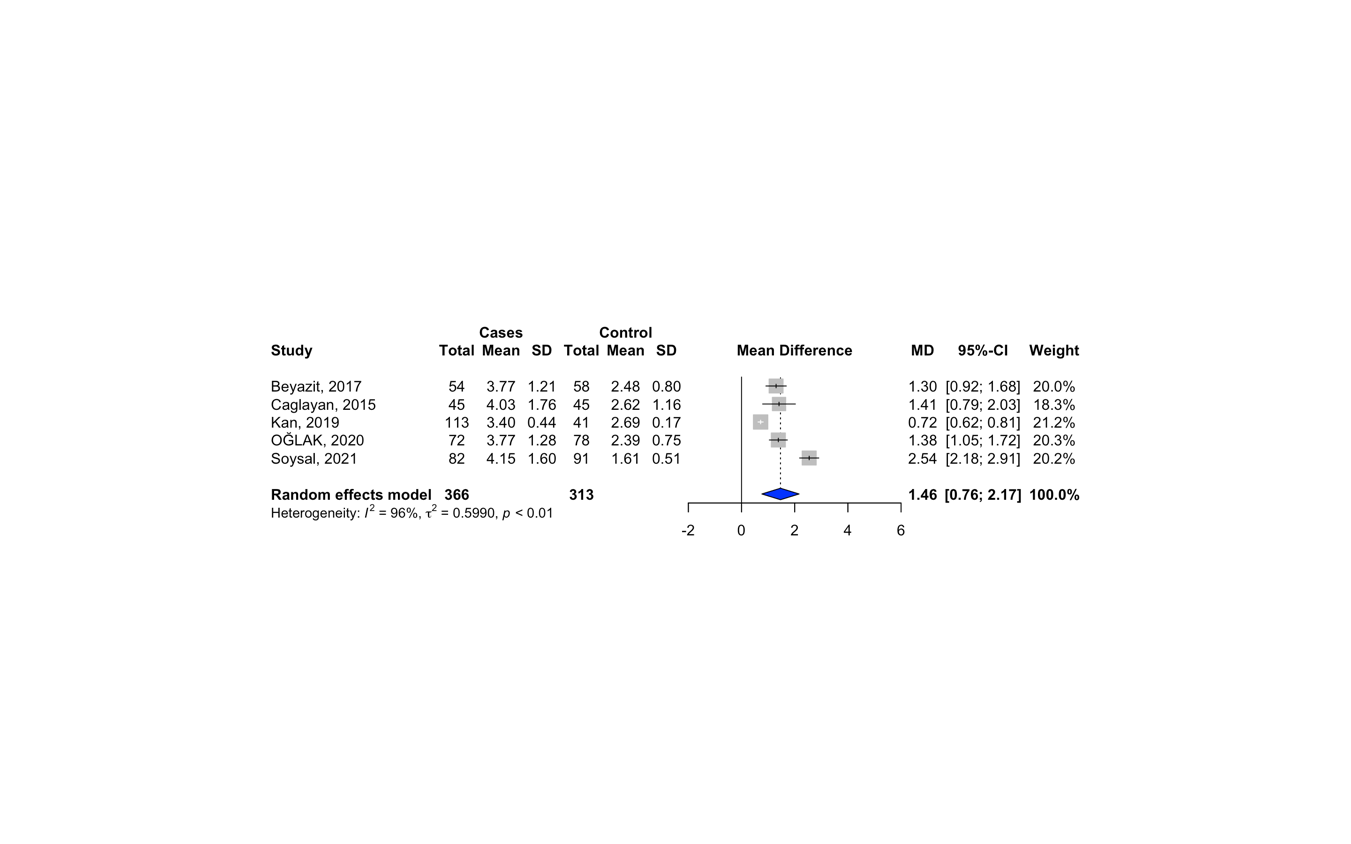


**Supplementary Material S7. Leave-one-out sensitivity analysis between patients with moderate and mild severity symptoms**

**
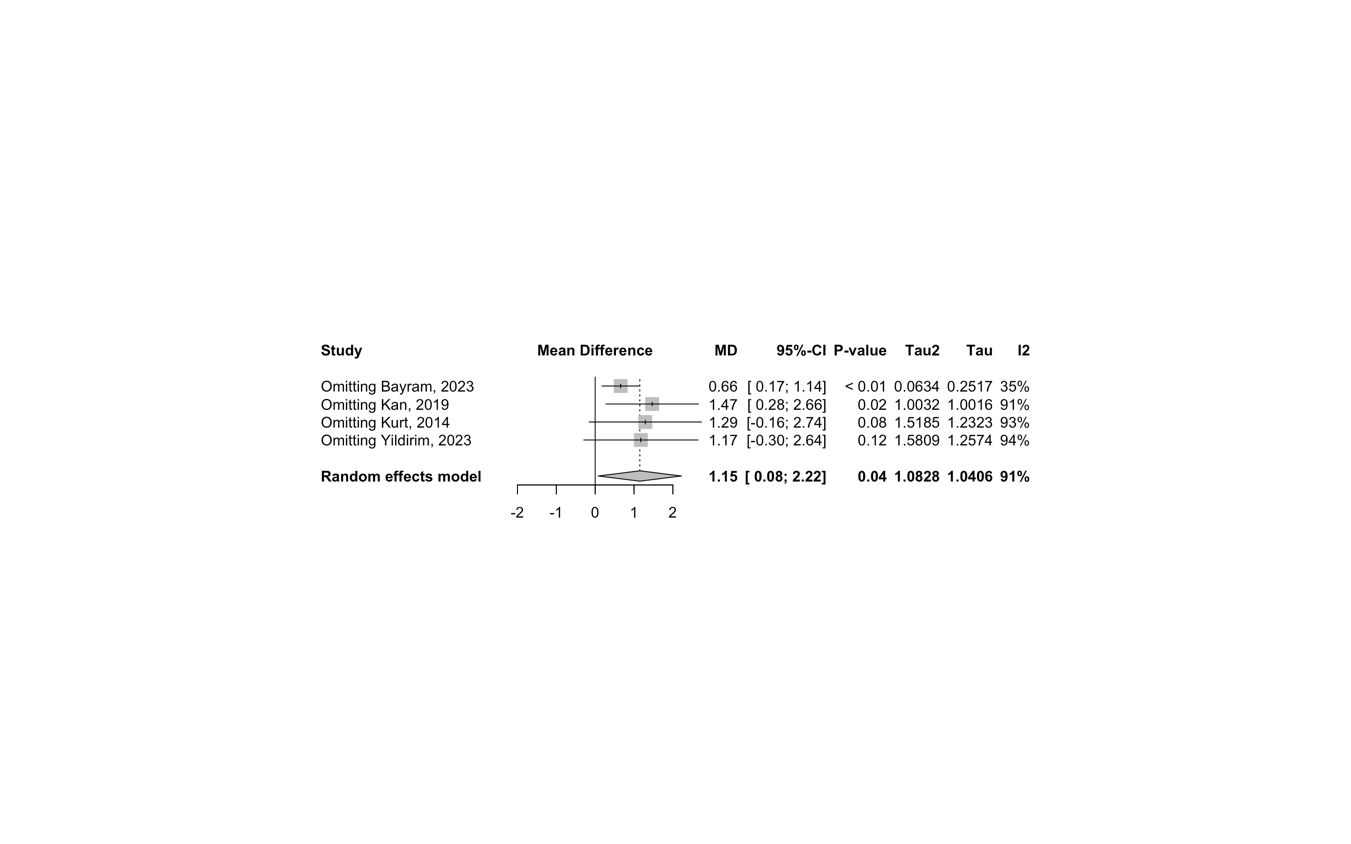
**

**Supplementary Material S8. Meta-analysis using Paule-Mandel estimator between patients with moderate and mild severity symptoms**


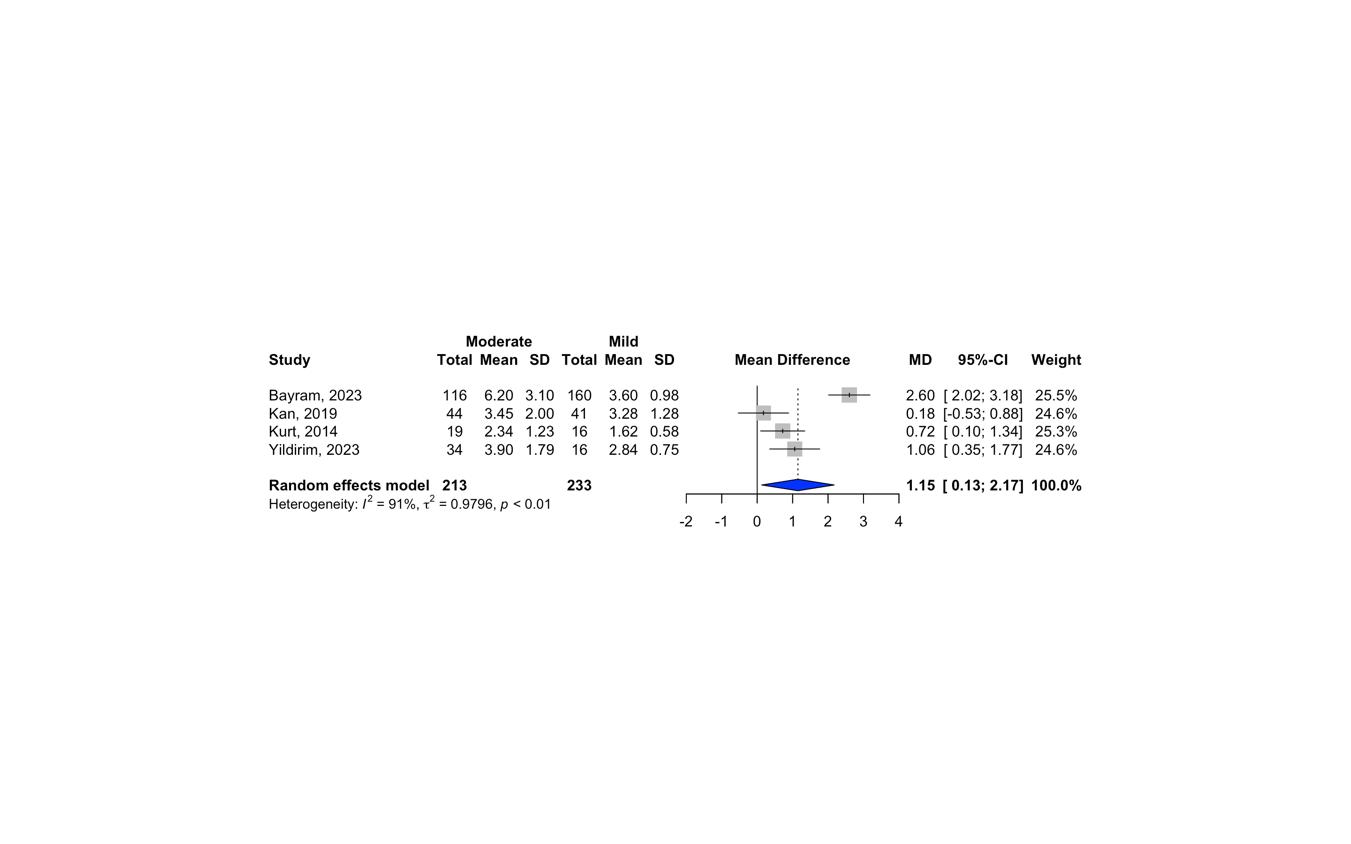


**Supplementary Material S9. Leave-one-out sensitivity analysis between patients with severe and mild symptoms**

**
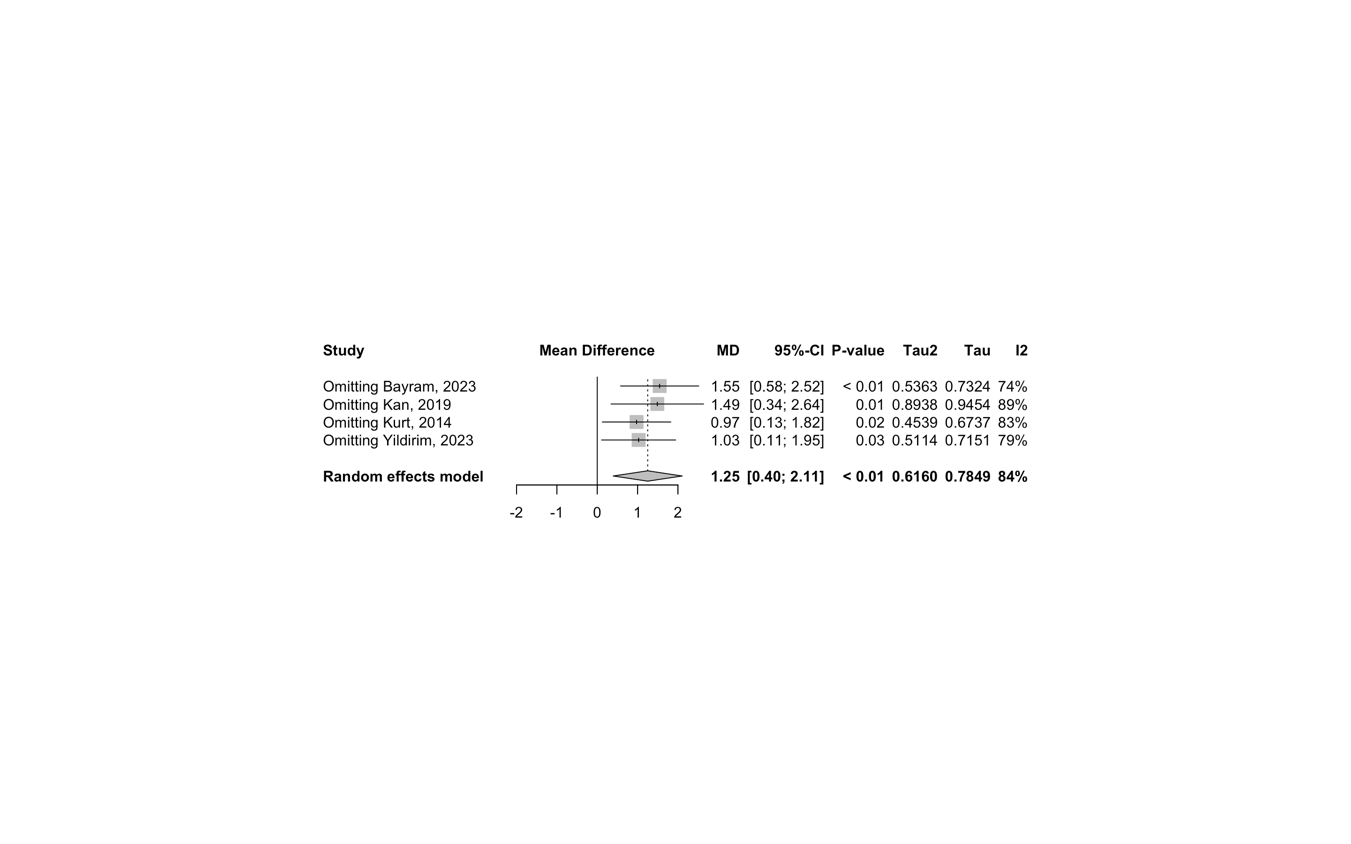
**

**Supplementary Material S10. Meta-analysis using Paule-Mandel estimator between patients with severe and mild symptoms**

**
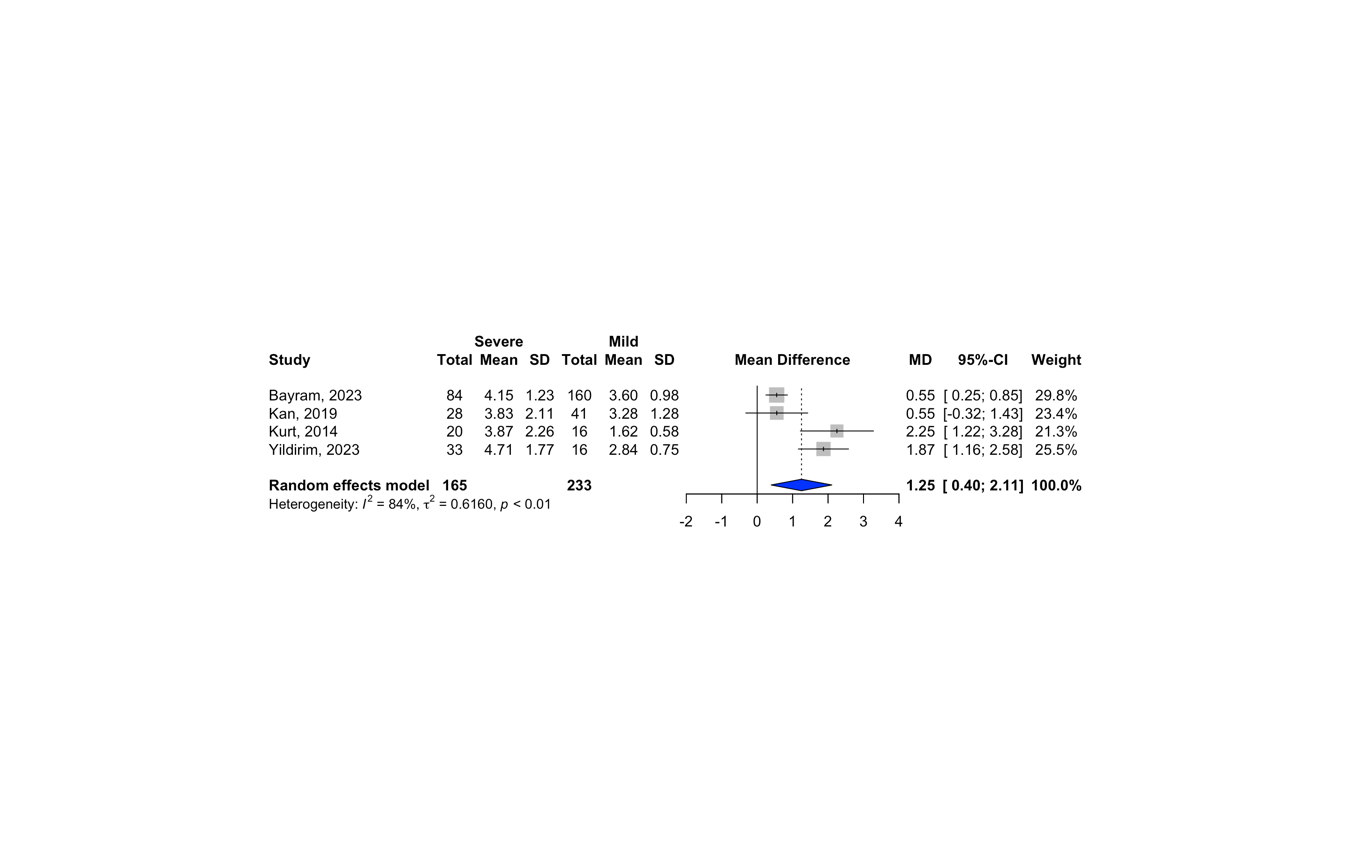
**

**Supplementary Material S11. Leave-one-out sensitivity analysis between patients with severe and moderate symptoms**

**
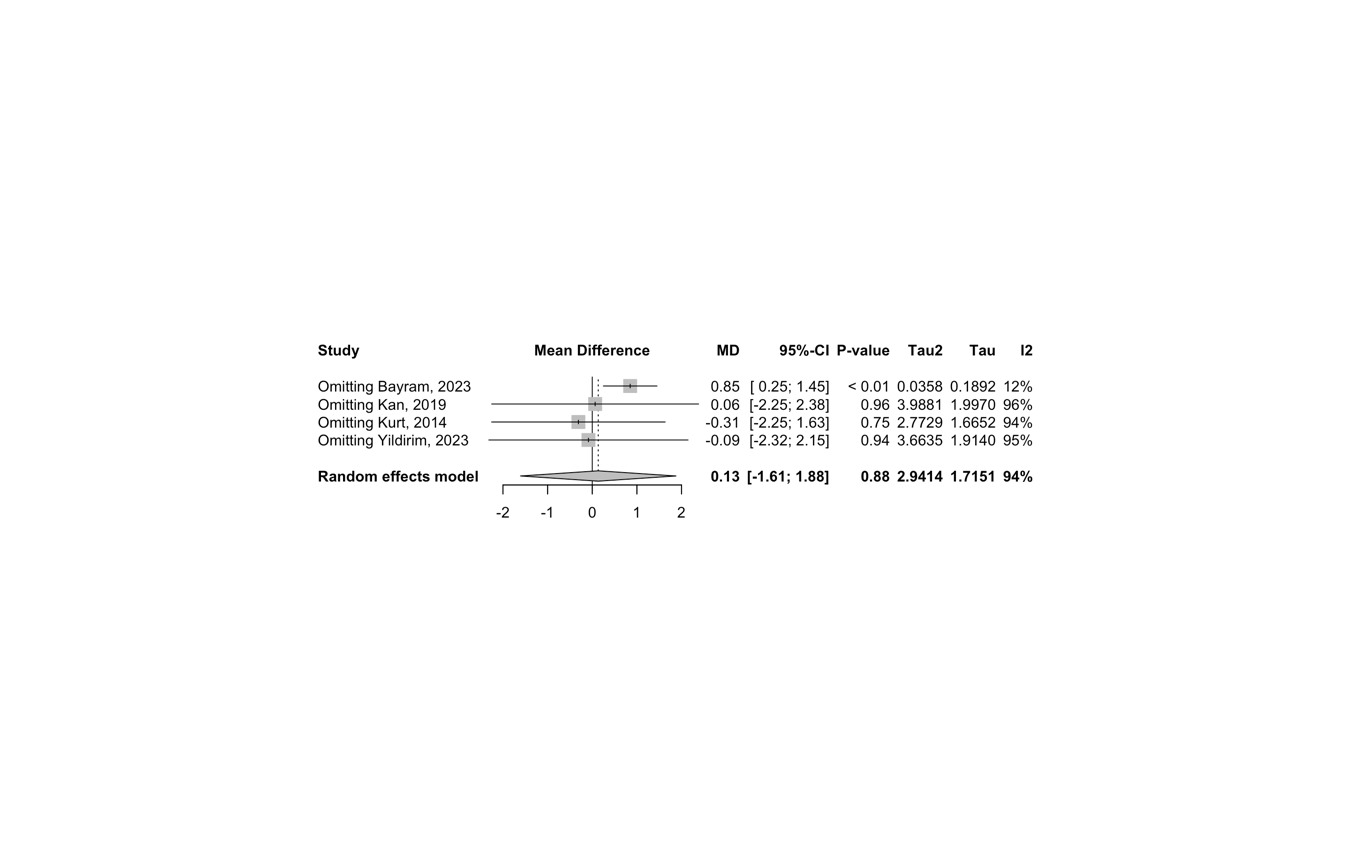
**

**Supplementary Material S12. Meta-analysis using Paule-Mandel estimator between patients with severe and moderate symptoms**

**
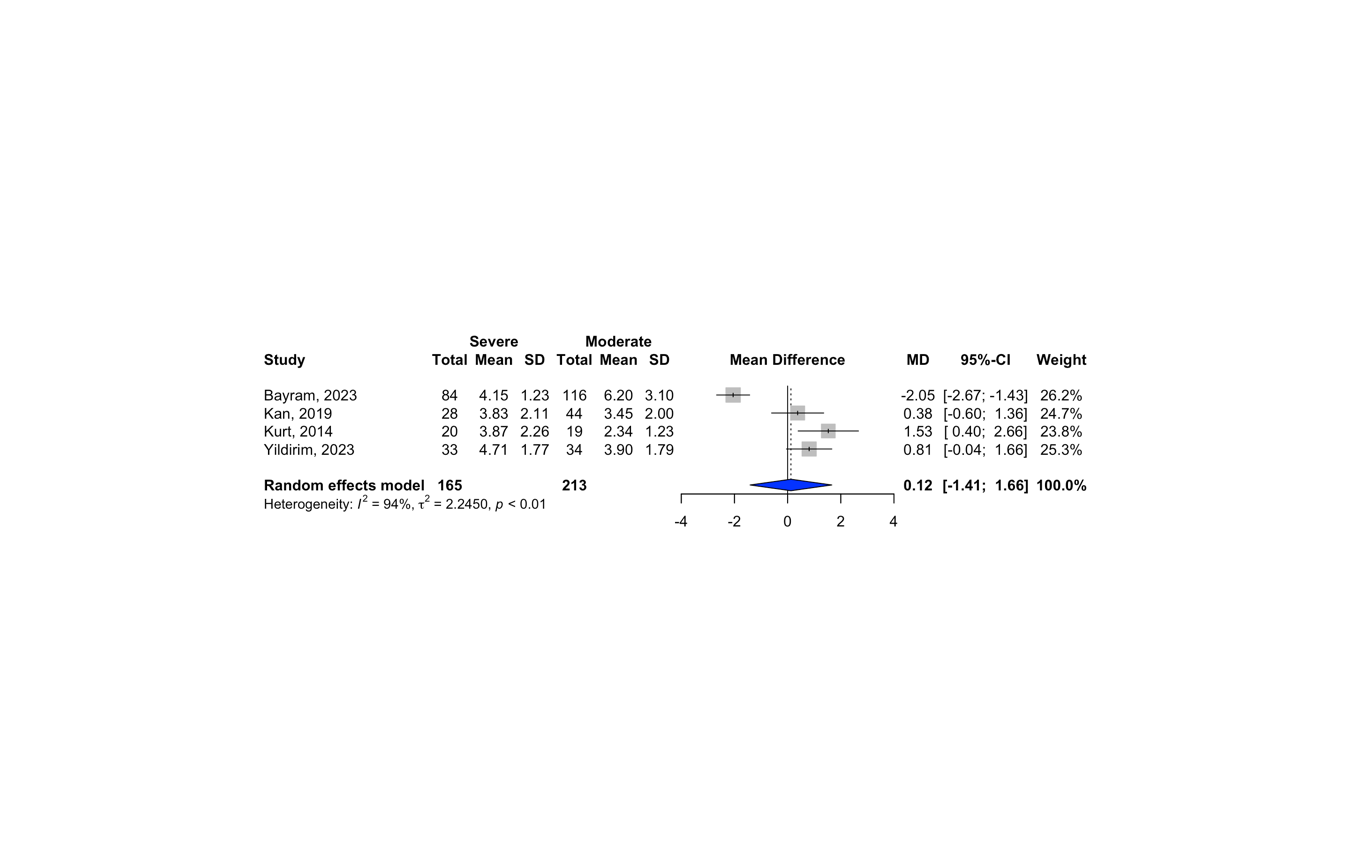
**

**Supplementary Material S13. Pooled results of Fisher´s Z transformed correlation between NLR and ketonuria levels**


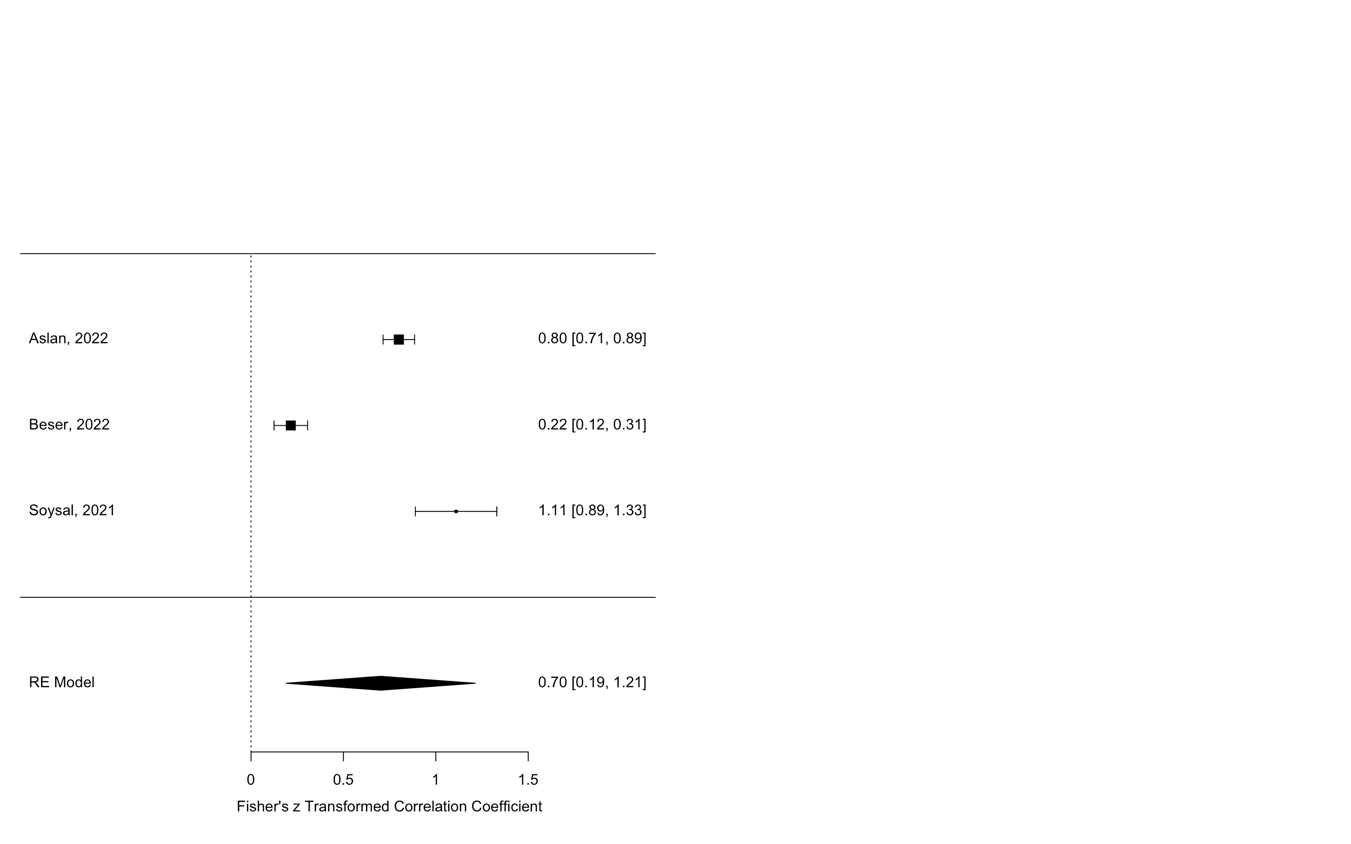


**Supplementary Material S14. Pooled results of Fisher´s Z transformed correlation between NLR and C-Reactive Protein**


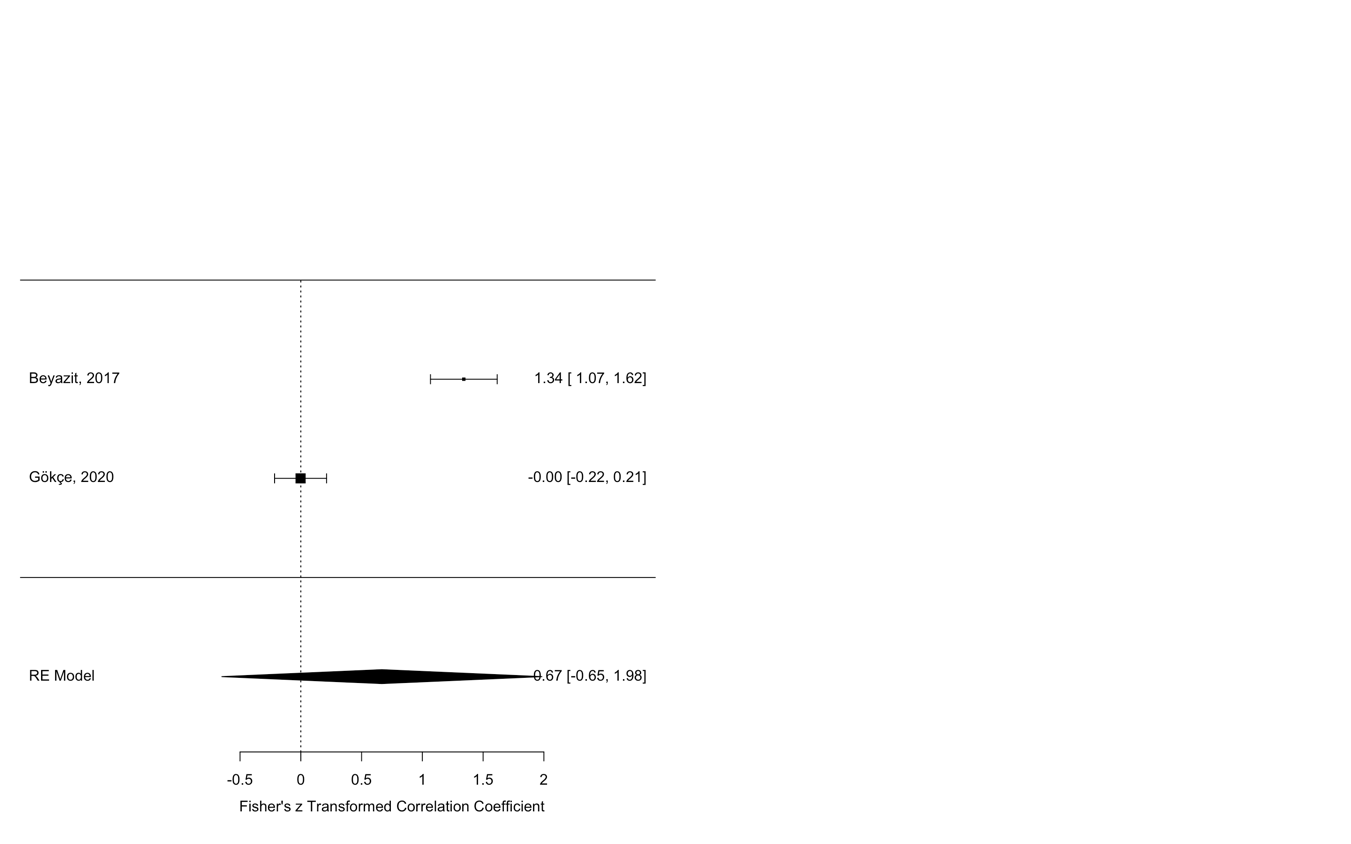


**Supplementary Material S15. Funnel plot about Hyperemesis gravidarum and healthy pregnant women**


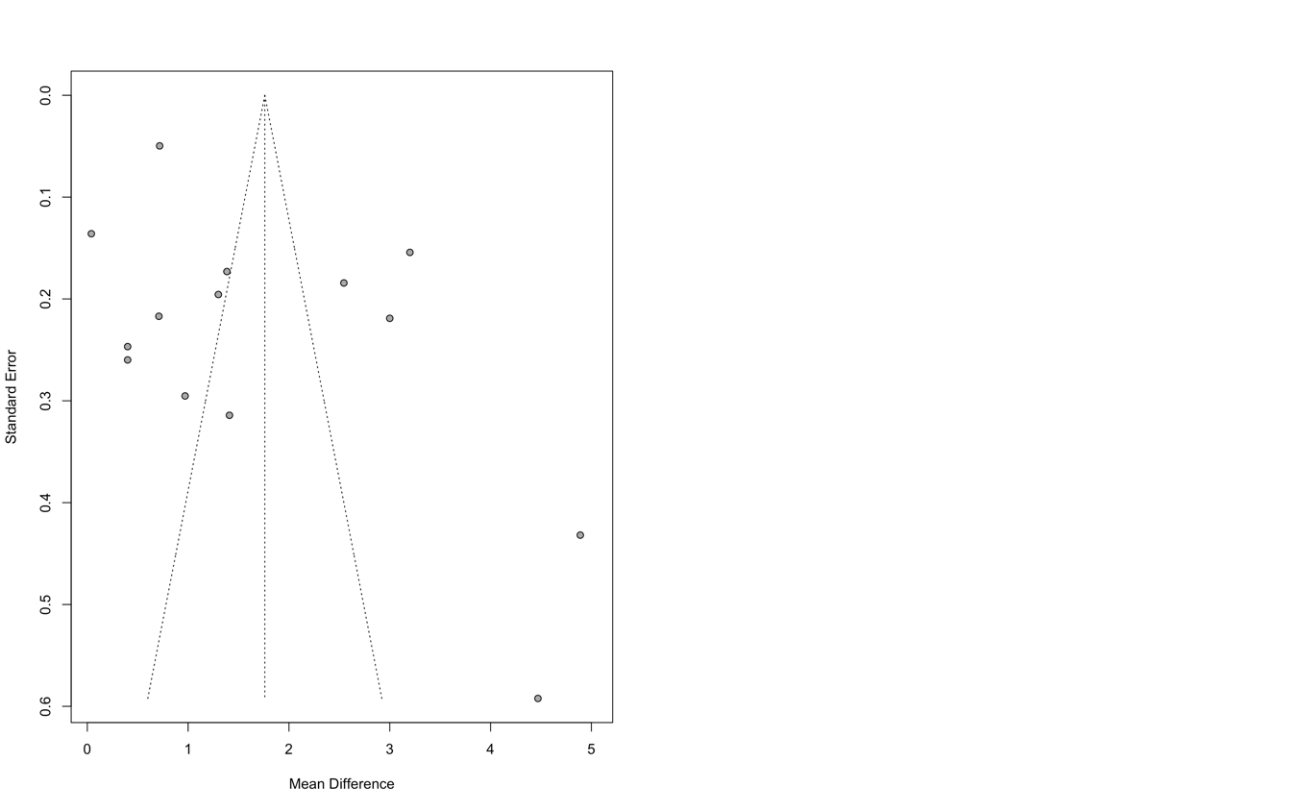

Supplement: Supporting Information — Additional supporting information can be found online in the Supporting Information section. S1–S15: All additional materials are available in this section. [file 4872025.f1.docx]
